# Supplementary material for: Sorting at embryonic boundaries requires high heterotypic interfacial tension
Source: Nat Commun. 2017 Jul 31;8:157. doi: 10.1038/s41467-017-00146-x (PMC5537356; doi:10.1038/s41467-017-00146-x)
Supplement: Supplementary file 2 — Supplementary Software 1 [file 41467_2017_146_MOESM2_ESM.zip › PottsModel/SrcPottsModel/doc/index-files/index-17.html]

R-Index


JavaScript is disabled on your browser.


Skip navigation links


- Overview
- Package
- Class
- Use
- Tree
- Deprecated
- Index
- Help

- Prev Letter
- Next Letter

- Frames
- No Frames

- All Classes

A B C D E F G H I J K L M N O P R S T U V W 


## R

readLattice(String) - Static method in class model.LatticeUtilities


readMatrix(String) - Static method in class utils.IO


readMatrix2(String) - Static method in class utils.IO


remove() - Method in class model.Lattice.PixelIterator


removeAllObservers() - Method in class mvc.AObservable


removeCell(Cell) - Method in class model.CellSet


removeCell(int) - Method in class model.CellSet


removeObserver(Observer) - Method in class mvc.AObservable


removePixel(Pixel) - Method in class model.Cell
:   Removes pixel from cell and readjusts center of mass, area and perimeter
    if it's not medium.

repaint(Lattice) - Method in class gui.SnapshotManager


repeatFrequency - Static variable in class engine.EnergyStatistic


reset() - Method in class engine.Utils.EnergyTracker


reset() - Method in class gui.ConfigurationInformationPanel


reset() - Method in interface gui.EngineObserverPanel


reset() - Method in class gui.PottsFrame


reset() - Method in class gui.StatusBar


resetIterator() - Method in class model.Lattice.PixelIterator


RGBToHSBColor(int, int, int) - Static method in class gui.Utils


rotate(Point, float) - Method in class model.FiniteVector


rotate(Point, float) - Method in class model.HalfLine


rotate(Point, float) - Method in interface model.Vector
:   Move the vector towards a point by a percentage of the angle between them.

run() - Method in class engine.Simulation


runSimulation() - Method in class engine.PottsEngine
:   Run simulation for given Constants once the PottsEngine has been
    initialized.

A B C D E F G H I J K L M N O P R S T U V W

Skip navigation links


- Overview
- Package
- Class
- Use
- Tree
- Deprecated
- Index
- Help

- Prev Letter
- Next Letter

- Frames
- No Frames

- All Classes
